# Structural and Molecular Characterization of *meso*-Substituted Zinc Porphyrins: A DFT Supported Study

**DOI:** 10.3390/molecules16129957

**Published:** 2011-12-01

**Authors:** Rudolf Słota, Małgorzata A. Broda, Gabriela Dyrda, Krzysztof Ejsmont, Giuseppe Mele

**Affiliations:** 1 Faculty of Chemistry, Opole University, ul. Oleska 48, 45-052 Opole, Poland; 2 Department of Engineering and Innovation, University of Salento, Via Arnesano, 73100 Lecce, Italy

**Keywords:** zinc porphyrins, molecular structure, DFT theoretical calculations, IR spectra

## Abstract

Structural parameters of a range of over 100 *meso*-substituted zinc porphyrins were reviewed and compared to show how far the nature of the functional group may affect the interatomic distances and bond angles within the porphyrin core. It was proved that even despite evident deformations of the molecular structure, involving twisting of the porphyrin's central plane, the coupled π-bonding system remains flexible and stable. DFT calculations were applied to a number of selected porphyrins representative for the reviewed compounds to emphasize the relevance of theoretical methods in structural investigations of complex macrocyclic molecular systems. Experimental and DFT-simulated IR spectral data were reported and analyzed in context of the individual molecular features introduced by the *meso* substituents into the porphyrin moiety base. Raw experimental spectral data, including ^1^H- and ^13^C-NMR, UV-Vis, FTIR, XRD, and other relevant physicochemical details have been provided for a specially chosen reference zinc porphyrin functionalized by *tert*-butylphenyl groups.

## 1. Introduction

The incessant scientific interest in porphyrins finds its origin in their affinity with chlorophyll and heme, the Nature-created metalloporphyrin species considered crucial to evolution of life on Earth. Hence, synthetic analogues featuring the characteristic porphyrin macrocycle have been expected to reveal a great application potential. Relative easy synthesis at moderate cost and a world of possibilities in creating of diverse structural modifications offer almost unlimited prospects for molecular design. The most important properties of the porphyrins are manifested first in their electronic absorption spectra, reflecting the unusual physicochemical nature of this peculiar class of compounds. Nevertheless, it is the character of the bonding setup which determines the specific chemistry of the porphyrin family. A comprehensive compilation entirely devoted to porphyrins can be found in the *Handbook of Porphyrin Science* [1].

In general, the chemistry of different metalloporphyrins is controlled by the complexed metal and the kind of peripherally and/or axially fixed substituents. These factors influence the electronic density distribution within the core of the macrocycle and thus determine its reactivity and stability, as well. Structural details are essential in learning the basic chemistry of such compounds. Therefore, crystallographic and molecular studies are of principal importance in the search for their unique properties.

**Figure 1:**
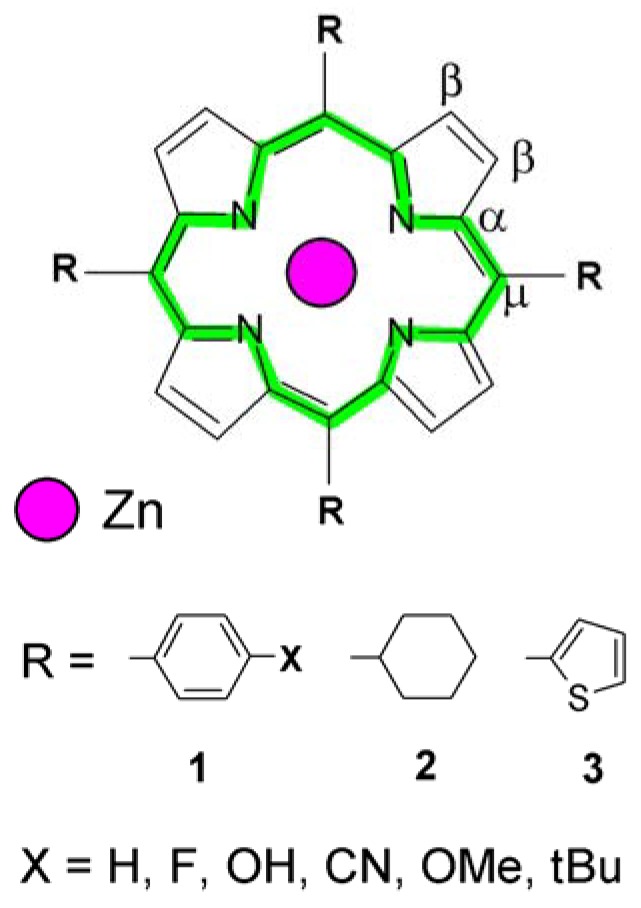
General structure of the tetra-*meso*-substituted Zn-porphyrins (OMe = methoxy; tBu = *tert-*butyl).

Natural porphyrins, e.g., the chlorophylls, have been substituted predominantly at the pyrrole periphery. Undoubtedly, the four carbon atoms (*meso* carbons) coupling the pyrrole units to form the characteristic macrocycle represent the weakest sites of the whole bonding system (Figure 1). They seem to be an easy target for a chemical attack particularly when reactive electron acceptors are present in the ambient. However, when substituted by bulky particles that could provide some kind of shielding, the strength of the porphyrin moiety may increase. Such structurally induced reinforcement is reflected, e.g., in considerably higher thermal stability of *meso*-substituted synthetic porphyrins [2,3], compared to the chlorophylls and similar analogues [4]. Depending on the substituents, the resulting structure of the porphyrin core may be more or less affected, thus making the compound more or less sensitive to the influence of the environment. Phenyl-derived radicals seem to be appropriate candidates for protecting the bonding set-up at the *meso* sites. Therefore, a wide range of them will be discussed below in the context of their potential influence upon the structural parameters of the porphyrin moiety.

**Figure 2:**
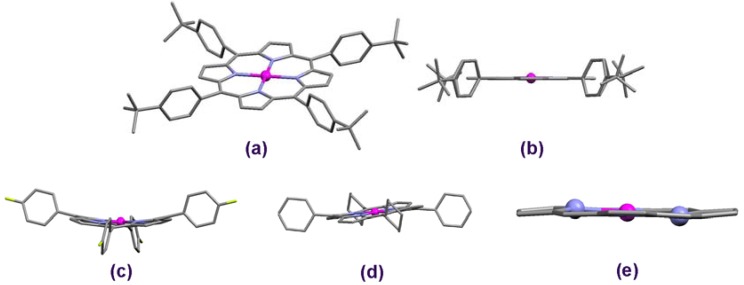
Molecular structure of diverse Zn-pophyrins (derived from crystallographic data). Structures (**a**) and (**b**) refer to ZnTBPP [7]; (**c**) Cyanophenyl analogue (R = 1, X = CN) [9]; (**d**) Cyclohexyl analogue (R = 2) [10]; (**e**) Bent pyrrole units of the porphyrin core, adapted from [11]; two opposite nitrogen atoms have been highlighted as blue balls to emphasize the distortion.

Porphyrins with all *meso* carbons equally functionalized by substituted phenyls belong to the most extensively explored ones within the last few decades and still remain an attractive research subject. Among them, those including the *tert*-butylphenyl groups have gained special attention (Figure 2). Particularly, they were found to demonstrate very interesting photochemistry, which is believed fundamental for the development of novel application areas. For instance, the Cu(II) porphyrin with *tert*-butylphenyl groups in either of the *meso* positions, CuTBPP, (where TPBP denotes the 5,10,15,20-tetrakis(4-*tert*-butylphenyl) porphyrin moiety, C_60_H_60_N_4_) revealed reasonable photocatalytic activity in composite sensitizer-TiO_2_ catalyst systems [5,6]. The identically functionalized ZnTBPP complex [7], which in the present work has been chosen as a reference model compound, is supposed to feature similar chemistry. Moreover, a range of TBPP metalloporphyrins revealed lipophilic properties and displayed diverse activity when embedded into liposome membranes [8]. This fact may be crucial to their potential application in biological systems.

Undoubtedly, structure-related chemical properties and the morphology of the crystalline solids are important factors, which may influence the behavior of a porphyrin-based system under specified circumstances considerably. In the present study over 100 crystal structures of Zn-porphyrins including diverse phenyl-based groups linked to the macrocycle via the *meso* carbons have been compared with each other. Particularly, the geometry of the bonding system within the porphyrin core was analyzed to find out how far this can be modified by the *meso*-substituents itself and/or the solvent species incorporated into the crystal lattice. This study was supplemented by theoretical molecular modeling and had been expected to provide a more profound insight into the nature of *meso*-substituted porphyrins. During the recent years DFT calculations became an important tool in analyzing structural and spectral properties even in the case of complex molecular systems like those represented by the porphyrin species [12,13,14]. Based on our previous experience [15], we have extended the theoretical approach by simulating the IR vibrational spectra for selected compounds, representative for the analyzed structures. In this way, to some extent, it was possible also to estimate the impact of the particular *meso*-functionalization on the dynamic behavior of the whole macrocyclic setup. Moreover, by comparing the experimental and DFT-derived results we intended to highlight the growing significance of theoretical calculations in contemporary physicochemical studies.

## 2. Results and Discussion

For the reason of this work, we chose a reference model porphyrin 5,10,15,20-tetrakis(4-*tert*-butylphenyl)porphyrinato zinc(II) (ZnTBPP), presented in Figure 1 (R = 1, X = *t-*Bu) and Figure 2. This particular compound was extensively studied by our group for its crystallographic structure and diverse physicochemical properties [7,8]. The ZnTBPP complex has been characterized in the Experimental section; however XRD powder diffraction records along with raw spectral data have been reported in the Supplementary Information appendix. From the single crystal measurements it has followed that the model compound ZnTBPP represents a very well established molecular system. The Zn atom is found in a plane defined by the four pyrrole-nitrogen atoms and the N-Zn-N bond angles are ca. 90°, showing ideal square-planar coordination. The macrocycle itself shows no apparent distortion and one may consider it as "perfectly flat" (Figure 2b). The *meso* substituted benzene rings are not perpendicular to the plane of the macrocycle, yielding dihedral angles of 78.4° and 79.2°. Average bond lengths related to the porphyrin macrocycle have been collected in Table 1. For a complete listing of bond distances and angles see the previous work [7]. This model structure was compared with crystal data acquired for a set of 105 selected structural analogues, derived from a crystallographic database available elsewhere [16,17,18,19] and corresponding to those shown in Figure 1 for R = 1 (*i.e.*, various phenyl-based groups); the relevant structural data have been collected in Supplementary information appendix. The study was principally dedicated to the influence of diverse *meso* substituents on the geometrical relations within the Zn-porphyrin macrocycle and essentially it was focused on the bonds constituting the inner core, *i.e.*, the coupled π-electronic system. For the selection of molecules as listed in Figure 1, the molecular structure was obtained also from DFT theoretical calculations. Versatile possibilities offered by the Gaussian 09 package [20] prompted us to calculate the IR spectra of these compounds and analyze the effect of the particular substituents on the frequency of bond oscillations within the porphyrin core. Moreover, animation of the individual vibrational modes simulating the dynamic behavior of the porphyrin moiety allowed relating it with the potential effect of the substituting groups, thus supplementing the results of structural investigations.

### 2.1. Molecular Effects in Real Crystals and DFT-Derived Zn-Porphyrins

Generally, the X-ray determined structure of ZnTBPP [7] fits well into the range of parameters previously reported for related Zn-porphyrins of similar molecular construction [16,17,18,19]. In addition, the values following from the theoretical model very well agree with the measured data, as reported in Table 1 and Table 2. This is an important result illustrating the significance and credibility of theoretical modeling in advanced molecular studies of porphyrins.

**Table 1:** Average interatomic distances within the pyrrole subunit, *meso*-bridges and Zn-N for the model compound ZnTBPP (R = 1, X = tBu) and diverse zinc porphyrin molecules (acc. to Figure 1); "crystal" applies to values calculated from real crystallographic data reported elsewhere and "DFT" refers to theoretical results obtained in this work. For R = 1 the data represent average values of 105 diverse molecules analyzed.

Porphyrin type		Zn-N	C_α_-N	C_α_-C_β_	C_β_-C_β_	C_α_-C_μ_
ZnTBPP (crystal)	[7]	2.04	1.38	1.44	1.36	1.40
ZnTBPP (DFT)		2.07	1.40	1.46	1.37	1.42
R = **1** (crystal)	[16,17]	2.04	1.38	1.44	1.35	1.40
R = **2** (crystal)	[10,21]	2.03	1.38	1.44	1.35	1.40
R = **3** (crystal)	[11]	2.02	1.36	1.43	1.33	1.38

**Table 2:** Core perimeter and average C_μ_-R distance in selected tetra-*meso*-substituted zinc porphyrins: Experimental (exp.) and DFT-derived results (all values in Å); porphyrin structures according to Figure 1; references to literature apply exclusively to experimental data.

Porphyrin type	References	Core perimeter (exp.)	Core perimeter (DFT)	C_μ_-R distance (exp.)	C_μ_-R distance (DFT)
R = H		-^1)^	22.37	-^1)^	1.09
R = **1**, X = H	[22]	22.22	22.50	1.50	1.50
R = **1**, X = F	[23]	22.14	22.49	1.48	1.50
R = **1**, X = OH	[24]	22.24	22.50	1.50	1.50
R = **1**, X = CN	[9]	22.24	22.49	1.50	1.50
R = **1**, X = OMe	[25]	22.28	22.50	1.51	1.50
^2)^ R = **1**, X = tBu	[7]	22.24	22.50	1.50	1.50
R = **2**	[21]	22.29	22.57	1.53	1.54
R = **3**	[11]	21.99	22.49	1.47	1.49

^1)^ crystal data not available; ^2)^ ZnTBPP.

Comprehensive analysis of available crystallographic data supported by a detailed literature study have proved that the ideal molecular symmetry, as observed in our ZnTBPP complex, may be more or less distorted in the case of diverse tetra-*meso*-substituted Zn-porphyrins reflecting the impact of the incorporated species and when crystallized from different solvents (Figure 2). Structures with the bent pyrrole rings are produced, e.g., when X = CN [9] or F [23], Figure 2c. Such "saddle" conformations usually result from interactions between the adjacent macrocycles within the crystal lattice, often involving the solvent species (solvent effect). This is particularly pronounced when bulky solvent molecules have been accommodated by the crystal unit, as found, e.g., in the case of a Zn-porphyin clathrate (R = **1**, X = Br) containing 4 mesithylene species per one of the complex [26]. Another interesting example is the pentakis-guaiacol clathrate (R = **1**, X = OH) characterized by a distinct discrepancy in interatomic distances, 5%–10% for the same bond type [24]. Oxygen- and/or chloride-containing solvents seem to produce structural disorder particularly for porphyrins substituted by R = 1 with X ≠ H, although some slight deviations may be observed even when X = H [22]. In the case of methoxybenzene solvates, the oxygen atom of the "methoxy" group was found to interact with the Zn atom slightly pulling it out from the plane defined by the four pyrrole nitrogens (R = **1**, X = F) [23], showing up a convex-shaped porphyrin core, unique among the other structures reviewed of the Cambridge Structural Database [16,17]. The bent pyrroles and bond length differences of 3%–5% have also been exhibited in molecules where R = 1 and X = *n*-octyl [27]. In this case the structural deviations have been generated presumably by the aliphatic substituents. Nevertheless, in each of these "distorted" molecules, the core perimeter was found close to 22.24 Å, *i.e.*, well within the range determined for all structures regarded, Table 2.

Additionally, the interatomic distances within Zn-porphyrins including R = **1** have been compared with those involving R = **2** and **3**, because of diverse volume of these *meso*-coupled species relative to R = **1**. The cyclohexyl radical (R = **2**, [10]) does not noticeable influence the size of the porphyrin core, although the macrocycle itself proves slightly twisted and the *meso* C_μ_-R bonds appear somehow longer (2%) than in the case of R = **1** (Figure 2d, Table 2). Almost flat macrocycle and bond lengths typical for the studied Zn-porphyrin family are demonstrated also for R = **3** [11]. However, in this case the pyrroles of the porphyrin core are slightly bent, as shown in Figure 2e, and this type of distortion was relative frequently observed among the reviewed Zn-porphyrins. The C_μ_-R distances are ca. 2% shorter than for ZnTBPP, because of the smaller volume of the thienyl radical. In fact, for all of the molecules considered in this survey, the average C_μ_-R distance is kept relative close to 1.50 Ǻ. Interestingly, for another bulky substituent, 3,5-di-*tert*-butylphenyl, the porphyrin core proved ideal planarity, despite considerable torsion of the C_μ_-R bonds (ca. 64°) and some differences in length (ca. 4%) of the C_β_-C_β_ periphery within the pyrrole units [28]. 

The existence of a very stable molecular core is a crucial conclusion to this analysis of diverse *meso*-substituted Zn-porphyrin structures. Regardless of R and X (Figure 1), there is no such tendency revealed, that the bonds at the *meso* carbons be considerable affected. In most of the analyzed structures [16,17], even when the pyrroles were bent or twisted, the C-N and C-C bonds constituting the coupled π-electronic system are supposed to retain their stability since they do not differ from each other for more than 2% in length. Indeed, this fact has been confirmed by the crystallographic datareported thus far and reflected in a very similar porphyrin core perimeter displayed by the compounds compared, as shown in Table 2. This resulted also from DFT calculations, and the theoretically derived porphyrin species were found to reveal almost identical structural features like those following from X-ray diffraction files. The difference between DFT results and the respective real crystal values amounted only 1.0%–2.3% (Table 2), which practically justifies the significance of molecular calculations. Although the DFT-calculated structures have been referred to vacuum conditions, the general features shown by the derived molecular models, such as deviations from core-planarity, matched well those observed in real crystals. For details concerning the calculated Zn-porphyrin structures see the Supplementary information appendix. Moreover, as follows from Table 2, the core dimensions in *meso*-substituted porphyrins do not differ from those determined for Zn-porphine (R = H, Figure 1). This may indicate that the *meso*-substituents by no means do weaken the macrocycle's strength. Incidentally, the almost equal C_α_-N and C_α_-C_μ_ bond lengths within the porphyrin core provide important evidence for a well organized and flexible bonding set-up and hence a very stable molecular system in tetra-*meso*-functionalized Zn-porphyrins. Thus, one may expect them to exhibit a considerable potential to prevent the porphyrin unit from degradation under unfavorable conditions. Indeed, the studied ZnTBPP complex appeared thermally resistant up to almost 540 °C (as determined from thermal analysis; see Experimental section). On the other hand the elastic molecular structure allows accommodating the energy of possible structural distortions which may determine the chemical activity of diverse zinc porphyrins, which appears particularly important in porphyrin-based catalysts.

### 2.2. Crystal Surface Structure

**Figure 3:**
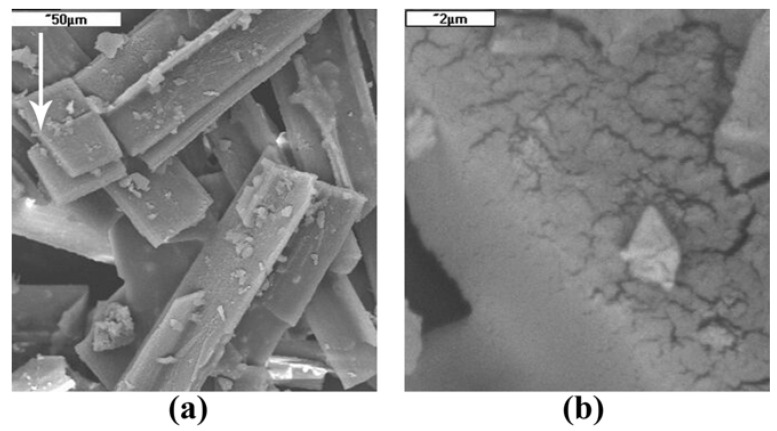
SEM micrographs of ZnTBPP crystals. (**a**) ×500; (**b**) ×10,000 (enlargement of arrow-pointed area).

To supplement the above considerations dedicated principally to the molecular structure, the morphology of solid crystals has been briefly addressed, too. Scanning electron microscopy (SEM) investigations performed for the model ZnTBPP compound proved the crystals were wrinkled and porous, as shown in Figure 3. Most probably the solid's interior consists of a network of fine pores, creating a "sponge-like" structure. This feature seems to be common for a large number of diverse phenyl-*meso*-substituted metalloporphyrin analogues, so-called *"porphyrin-sponges"* [9,22,25]. Presumably, this is also the reason for a rather fragile construction of single crystals and the problems met while trying to grow large mono-crystals suitable for X-Ray measurements. On the other hand, however, such well-developed microstructure should offer enhanced sorption activity, particularly important in catalytic systems. Moreover, the studied material revealed surface-adsorbed oxygen, as determined by the X-ray microprobe analysis (EDAX). This finding has not been surprising however, and results from the peculiar affinity of porphyrin analogues to molecular oxygen [29].

### 2.3. Infrared Spectra and Structure-Related Effects Based on DFT Calculations

External groups attached at the *meso*-carbon atom of the porphyrin core might have been suspected to induce some changes in electronic density distribution over the C_α_-C_μ_-C_α_ bridges, which eventually could have somehow affected the quality of these particular bonds. Although the structural analysis did not indicate for significant alterations in bond length, however the dynamic behavior of the macrocyclic bonding system could not be fully evaluated when exclusively based on crystallographic and theoretical molecular data. Therefore, the frozen-structure-related part of this work was supported by an infrared vibrational study to get some more profound insight into the porphyrin system modified by its functionalization at the bridging carbons (C_μ_).

**Figure 4:**
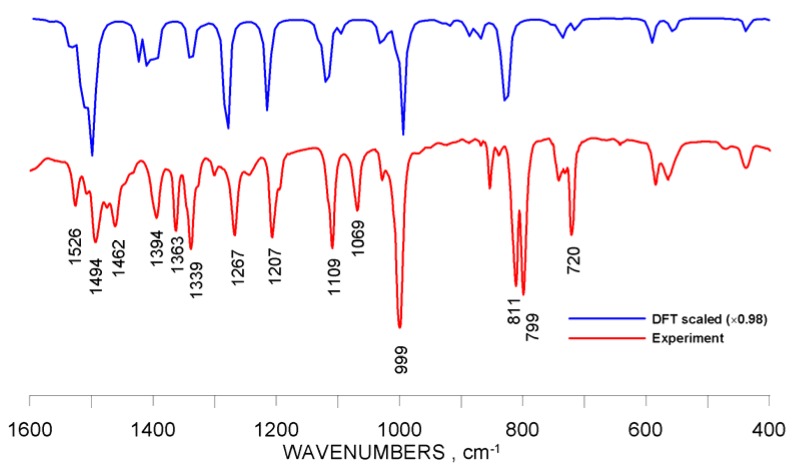
Experimental FTIR spectrum of ZnTBPP and the simulated IR spectrum obtained from DFT calculations.

The impact of the *meso*-substituents on the IR oscillations of bonds constituting the porphyrin core was examined for the model ZnTBPP compound and the selected set of diverse analogues shown in Figure 1. The particular active vibrational modes were identified based on DFT molecular calculations and using the animation feature of the GaussView 5.0 software. Besides, the FTIR spectrum measured for ZnTBPP was confronted with the DFT-simulated one (Figure 4). Additionally, the spectra calculated for Zn-porphine (R = H), tetraphenyl (R = 1, X = H) and tetrahydroxyphenyl (R = 1, X = OH) derivatives were compared with experimental FTIR data reported elsewhere, [14,30,31], respectively (see Supplementary Information appendix).

Comparison of the DFT-calculated spectra with the experimental results obtained both in this work and reported elsewhere basically indicates for common features of the spectrum patterns, although the peak values (frequency/intensity) in some cases differ from each other. Nevertheless, as shown in Figure 4, the scaled theoretical and real-measurement frequency values seem close enough to consider the DFT-derived IR spectral data important in advanced molecular studies. This assumption has found a strong backup in the relevant papers [12,13,32].

The oscillating porphyrin macrocycle actually represents a system of mutually coupled vibrations. Hence it is rather difficult to assign the frequency of the particular bands identified in the IR spectra to a localized oscillation mode. It proved possible (to a reasonable extent) extracting from the oscillating π-electronic setup those vibrations which refer to stretching of the C_α_-C_μ_-C_α_ bridges and these vibrations were used in further considerations. More tentatively however, the pure stretching vibrations of the C_μ_-R bonds could be separated from other oscillations manifested by the functional group (R). The computed frequencies related to the above-mentioned vibrations have been reported in Table 3.

**Table 3:** Calculated frequencies (ν) of IR stretching vibrations (not scaled) assigned to the C_α_-C_μ_-C_α_ bridges and (tentatively) to C_μ_-R oscillations for diverse *meso*-substituted Zn-porphyrins (according to Figure 1). The first-row frequencies (***bold italic*** font) apply to coupled diverse vibration modes, C_α_-C_μ_ and C_β_-C_β_ (see also Table SI-1, Supplementary Information appendix).

ν, cm^−1^									
Oscillation mode	R = H	R = 1 and X as below						R = 2	R = 3
		H	F	CN	OH	OMe	tBu		
C_α_-C_μ_	*1557*	*1565*	*1566*	*1568*	**1565**	*1565*	*1565*	*1575*	*1562*
		1539	1544	1546	1547	1546	1549	1533	1538
	1597	1520	1527	1531	1529	1529	1531		
C_μ_-R	3204	1648	1649	1655	1661	1658	1658	1414	1603
		1254	1253	1254	1254	1254	1258	1179	1191

**Figure 5:**
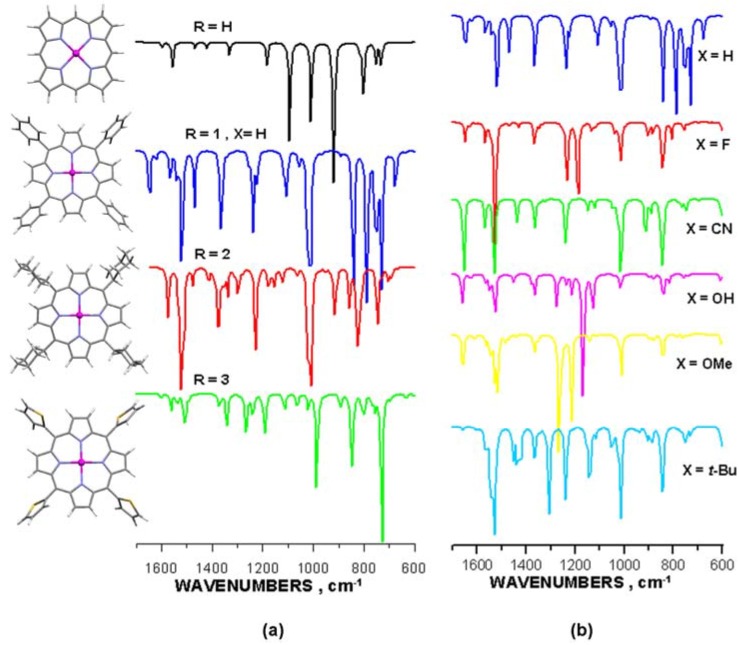
Infrared spectra calculated for selected Zn-porphyrins (raw results, not scaled). (**a**) Effect of the *meso*-substituent type. General molecular structure is shown; (**b**) Effect of the X-group in phenyl-*meso*-substituents.

The effect of the diverse *meso*-substituent type on the character of the IR spectra was illustrated in Figure 5a. On the other hand, the influence of the phenyl-derivatives most frequently used to substitute the *meso*-carbons of the porphyrin moiety was shown in Figure 5b. Full-range IR spectra derived from DFT theoretical calculations along with a listing (Table SI-1) of oscillation frequencies for vibrations basically related to the porphyrin macrocycle have been presented in the Supplementary information appendix.

In principle, the IR spectra conform very well to the crystallographic data analyzed in the preceding section and do supplement the conclusions drawn from structural considerations. Common features observed in the spectra displayed in Figure 5a essentially apply to the pyrrole unit of the macrocycle and may be assigned to the stretching of the C_β_-C_β_ bonds (1557–1575 cm^−1^), *"pyrrole-breathing"* oscillations (988–1018 cm^−1^) and C_β_-H *in plane* (1330–1370 cm^−1^) and/or *out-of-plane* (820–850 cm^−1^) vibrations (Table SI-1). On the other hand, the most apparent differences, which one may see in the spectra, follow from oscillations of the bonding system of the *meso*-substituents, R (see also the full-range spectra in Supplementary information). However, a closer look at the spectra has revealed some details evidently reflecting the impact of the functional group on the macrocycle's electronic system, due to different molecular volume and chemistry of the particular substituents (Table 3). Surely, the subtle distinction in frequency of the pyrrole-related oscillations one may have linked with the nature of the species attached at the *meso*-C atoms. The stretching vibrations of the C_α_-C_μ_ bonds characterize the possible electronic distribution in the vicinity of the *meso*-carbons, considered the most "sensitive" part of the porphyrin core and therefore, to some extent, the flexibility and strength of the macrocycle. Relatively similar values obtained for the phenyl-, cyclohexyl- and thienyl- analogues suggest that these groups induce a comparable molecular effect upon the porphyrin core. Only the frequency of C_α_-C_μ_ vibrations in the case of Zn-porphine (R = H) stands out of the row (Table 3), thus probably reflecting the lack of *meso*-substituted carbons. Conversely, stretching vibrations of the C_μ_-R bonds are featured by very different frequencies depending on the type of R. Definitely the least values were demonstrated by the cyclohexyl-derivative, which probably indicates for a weaker C_μ_-R connection. Obviously, the C_μ_-H stretching was found to be far out of the range determined for the bulky substituents (R = **1**, **2** and **3**), at 3204 cm^−1^. Besides, the oscillation mode of the C_μ_-R bonds proved to follow the vibrations observed for the functional group itself. Therefore the frequencies assigned to such seemingly localized vibrations are supposed to be treated with particular caution. In fact, they might have been reflecting rather the nature of the oscillating bonding system of the *meso*-substituent and not that of the C_μ_-R bond.

The phenyl-substituted porphyrins (R = **1**, X, Figure 1) display practically very similar spectral features in the part related merely to the macrocycle, as follows from Figure 5b and Table 3. Almost equal frequencies of the C_α_-C_μ_ vibrational modes as well as of those assigned to the pyrrole unit suggest the π-electronic system of the porphyrin core is not affected by the peripheral X substituents. The same conclusion applies when regarding the C_μ_-R stretching vibrations. Presumably, there might be some very subtle infrared-sensitive core-effects generated by the diverse phenyl-substituents, however these are of little importance and usually are not to be seen in the relevant IR spectra due to low intensity of such oscillations.

## 3. Experimental

### 3.1. Materials

*5*,*10*,*15*,*20-Tetrakis(4-tert-butylphenyl) porphyrinato zinc(II)* (ZnTBPP) was synthesized according to a typical procedure reported elsewhere [5], using ZnCl_2_ as the metal source. Pure monocrystals for crystallographic measurements were obtained by multiple re-crystallization from toluene (reagent grade) at room temperature, yielding the final product in the form of a toluene solvate. This complex was used in this study as a reference model zinc porphyrin and it has been characterized as follows: UV-vis (toluene): λ_max_, nm (log ε) 425 (5.40), 551 (4.05), 591 (3.36); FT-IR (KBr): ν, cm^−1^ 3029 (w), 2960 (s), 2903 (m), 2867 (m), 1526 (w), 1496 (m), 1462 (m), 1394 (m), 1363 (m), 1339 (m), 1267 (m), 1207 (m), 1109 (m), 1069 (m), 999 (vs), 811 (s), 799 (s), 720 (m); ^1^H-NMR (400 MHz; CDCl_3_; Me_4_Si): δ_H_, ppm 1.62 (36H, s, -CH_3_), 7.76 (8H, d, Ph), 8.15 (8H, d, Ph), 8.98 (8H, s, pyrrole-*H*); ^13^C-NMR (400 MHz; CDCl_3_; Me_4_Si): δ, ppm 29.66, 31.68, 121.11, 123.39, 131.90, 129.00, 134.28, 139.81, 150.26; thermal analysis (DSC): endoergic peak at 538.9 °C, DTG peak at 537.4 °C (92% loss of mass) corresponding to the compound decomposition. Electronic absorption and emission spectra (UV-Vis), ^1^H-NMR and FTIR raw spectra have been included in the supplementary information appendix.

### 3.2. Computational Methods

Density functional theory (DFT) method was applied to calculate the molecular structures and infrared spectra of selected porphyrins (Figure 1). The appropriate Z-matrices have been provided in the supporting information appendix. All calculations were performed using the Gaussian 09 software [20]. Geometry optimizations and frequency analysis for all studied compounds were carried out using the hybrid density functional B3LYP [33,34] and the LANL2DZ basis set [35]. The B3LYP functional has proved remarkable accuracy in many real-world problems [36,37] and has been considered probably the best compromise between speed and accuracy for relatively large systems such as functionalized metalloporphyrins or metallophthalocyanines. This was demonstrated in our recently published works [8,15].

### 3.3. Instrumental Methods

#### 3.3.1. X-ray Powder Diffractometry

Diffraction pattern of the pulverized polycrystalline material, ZnTBPP, was recorded on a Philips PW1710 diffractometer using CuK_α_ radiation (λ_α1_ = 1.54060; λ_α2_ = 1.54439 Ǻ). Complete set of powder XRD data is provided as supplementary material (see Electronic Supplementary data).

#### 3.3.2. Scanning Electron Microscopy (SEM)

Investigations were carried out by a JEOL JSM-5400 scanning electron microscope provided with an energy dispersive X-ray analyzer (EDAX). Prior to testing, the samples were evacuated (298 K, 100 Pa) and gold coated.

#### 3.3.3. Electronic Absorption Spectroscopy

UV-VIS spectra were measured by the means of a UNICAM 310 spectrophotometer within the range of 190–1100 nm, in a 10 mm quartz cuvette, at constant temperature 20 °C (thermostated).

#### 3.3.4. Electronic Emission Spectroscopy (Fluorescence)

Fluorescence spectra were recorded in toluene for λ_exc_ = 360 nm, using Perkin-Elmer MPF-3 spectrofluorimeter. Fluorescence quantum yield, Φ_F_, was determined by a comparative method using [Ru(bpy)_3_]Cl_2_ as standard.

#### 3.3.5. FTIR Spectroscopy

Philips FTIR PU 9800 spectrometer was used to measure the FTIR spectrum and the zinc porphyrin sample (ZnTBPP) was analyzed in a KBr disc.

#### 3.3.6. NMR Spectroscopy

^1^H-NMR and ^13^C-NMR spectra were recorded on a Bruker Avance II 400 Hz spectrometer.

#### 3.3.7. Thermal Analysis (DSC, DTG)

Universal V3.0G device (TA Instruments) was used to register the DSC (Differential Scanning Calorimetry) and TGA (Thermogravimetry Analysis) runs, under constant nitrogen flow (40 mL N_2_/min) and at a heating rate of 10 deg/min.

## 4. Conclusions

The bonding system in tetra-*meso*-substituted zinc porphyrins constitutes a very stable and flexible molecular set-up. Both the crystallographic and IR investigations have indicted that the considered *meso*-substituents practically do not affect the strength of the porphyrin core. DFT theoretical investigations appeared very useful in analyzing both structural and IR-spectral properties. It must be emphasized, that the IR spectra reported in this work well conform to the quoted references concerning both the experimental data and theoretically derived IR vibrational frequencies. Diverse structural effects observed in real Zn-porphyrin crystals may appear crucial to the catalytic activity of such compounds. The flexible porphyrin core allows the molecule to accumulate extra energy resulting from intermolecular interactions, which could even produce structural deformations but not disruption of the bonding system. Thus, under certain circumstances one may expect such compounds to exhibit enhanced reactivity. Therefore structural investigations are of principal meaning particularly in molecular designing of porphyrin solids considered for application in heterogeneous systems.

## Supplementary Material

Supplementary material can be accessed on: http://www.mdpi.com/1420-3049/16/12/9957/s1.
